# Interactions between hydatid cyst and regulated cell death may provide new therapeutic opportunities

**DOI:** 10.1051/parasite/2019070

**Published:** 2019-11-29

**Authors:** Sirous Mehrani Moghaddam, Stephane Picot, Ehsan Ahmadpour

**Affiliations:** 1 Immunology Research Center, Tabriz University of Medical Sciences 5166/15731 Tabriz Iran; 2 Student Research Committee, Tabriz University of Medical Sciences 5166/15731 Tabriz Iran; 3 Malaria Research Unit, SMITh, ICBMS, UMR 5246 CNRS INSA CPE University Lyon 69100 Lyon France; 4 Institute of Parasitology and Medical Mycology, Croix-Rousse Hospital, Hospices Civils de Lyon 69004 Lyon France; 5 Infectious and Tropical Diseases Research Center, Tabriz University of Medical Sciences 5166/15731 Tabriz Iran; 6 Department of Parasitology and Mycology, Faculty of Medicine, Tabriz University of Medical Sciences 5166/15731 Tabriz Iran

**Keywords:** Echinococcosis, Hydatid cyst, Regulated cell death, Apoptosis, Necrosis, Autophagy

## Abstract

Cystic echinococcosis and alveolar echinococcosis are chronic zoonotic infections, transmitted throughout the world. Development of the cestode larval stages in the liver and lungs causes damage to intermediate hosts, including humans. Several pathways leading to the suppression of host immune response and the survival of the cysts in various hosts are known. Immune response modulation and regulated cell death (RCD) play a fundamental role in cyst formation, development and pathogenesis. RCD, referring to apoptosis, necrosis and autophagy, can be triggered either via intrinsic or extrinsic cell stimuli. In this review, we provide a general overview of current knowledge on the process of RCD during echinococcosis. The study of interactions between RCD and *Echinococcus* spp. metacestodes may provide in-depth understanding of echinococcosis pathogenesis and open new horizons for human intervention and treatment of the disease.

## Abstract (fr)

L’échinococcose kystique et l’échinococcose alvéolaire sont des infections zoonotiques chroniques, transmises dans le monde entier. Le développement des stades larvaires des cestodes dans le foie et les poumons provoque des lésions chez les hôtes intermédiaires, y compris les humains. Plusieurs voies menant à la suppression de la réponse immunitaire de l’hôte et à la survie des kystes chez divers hôtes sont connues. La modulation de la réponse immunitaire et la mort cellulaire régulée (MCR) jouent un rôle fondamental dans la formation, le développement et la pathogenèse du kyste. La MCR, faisant référence à l’apoptose, à la nécrose et à l’autophagie, peut être déclenchée par des stimuli intrinsèques ou extrinsèques. Dans cette revue, nous fournissons un aperçu général des connaissances actuelles sur le processus de la MCR au cours de l’échinococcose. L’étude des interactions entre les métacestodes d’*Echinococcus* spp. et la MCR pourrait permettre d’approfondir la compréhension de la pathogénie et d’ouvrir de nouveaux horizons pour l’intervention humaine et le traitement de l’échinococcose.

## Introduction

Cystic echinococcosis (CE) and alveolar echinococcosis (AE) are zoonotic infections caused by the larval stages (metacestodes) of parasitic cestodes *Echinococcus granulosus* and *E. multilocularis* [15, 17, 52]. Metacestodes are able to persist in intermediate hosts for a long period of time (over decades), without causing obvious pathologic damage in host tissues [52]. The most dangerous complication of echinococcosis is cyst perforation, leading to death from septic shock or embolic complications [2, 47, 57].

**Figure 1:**
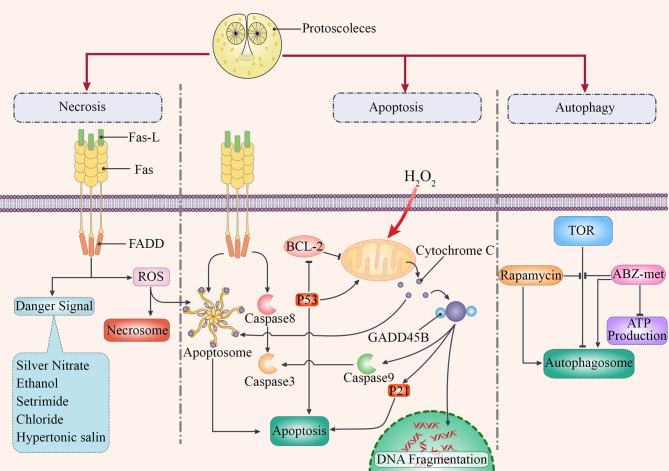
A summary of the potential mechanisms of cell death induced by *Echinococcus* spp. larval stage. The main molecules involved in cell death are shown, and the three main paths are shown with bold arrows. Directions for stimulation of necrosis, apoptosis and autophagy are shown. In the process of cell death caused by *Echinococcus* spp., apoptosis plays an important role in the cytolysis of the involved tissues. They can activate both extrinsic and intrinsic pathways. For example, some of them motivate mitochondria to release cytochrome C and P53, resulting in the formation of apoptosomes and activation of Caspase-3.

During CE, the cyst is created in the course of parasite maturation. Additionally, parasites can survive by developing cellular interactions with host tissues [51]. These specific interactions are probably facilitated by genetic heritage in the parasite from its mammalian hosts [6, 31, 50]. Chronic infections caused by the parasite depend on rich crosstalk with the host, which leads to an appropriate immune response and to the survival of both the host and the parasite [49] (Fig. 1).

Apoptosis of *E. granulosus* protoscoleces was first identified more than a decade ago by a group of researchers from Chile [33]. The link between DNA repair via RAD9 and cyst infertility was documented in the same laboratory in 2008 [7]. Researchers from China and Iran later confirmed the involvement of apoptosis in hydatid cyst pathophysiology [18, 51]. This last study opens the discussion on a bi-functional effect in the relationship between host and parasite, leading to a complex process of cell death regulation during hydatid cyst development. It was shown that extracted hydatid cyst fluid (HCF) induced lymphocyte apoptosis *in vitro*, demonstrating a direct effect of the parasite on host immune cells. This crosstalk has opened new treatment opportunities in the last decade [1, 3, 5, 8, 10, 18].

The first approach is to consider the early development of a cyst from approximately 10 stem cells located inside an activated oncosphere in the liver, as a process mimicking cancer [21, 42]. Based on that assumption, one could speculate that anti-cancer therapeutic approaches, including radiotherapy, should be tested to induce regulated cell death (RCD) of the parasite [25]. The second option is to address the issue of pro-apoptotic effects of HCF produced during infection of the intermediate host, and to test the hypothesis of potential value of certain specific compounds of this fluid as therapeutic agents for human cancers, including melanoma and breast cancer [11, 14, 16, 39, 51]. These two approaches may open an extensive research area, provided preliminary results obtained to date are consolidated and confirmed in large controlled studies.

## Regulated cell death of the *Echinococcus* parasite

Different proteins involved in a large array of cellular mechanisms, including cell survival, have recently been described at different stages of metacestode development [12, 48]. While there is no definitive evidence that the presence of these proteins may be linked to an effect on the RCD of the parasite, this paves the way for an interesting approach in parasite biology.

Prohibitin is a highly conserved protein involved in multiple functions depending on its localization, and is crucial for apoptosis processes in human cells [37]. *E. granulosus* prohibitin (EgPHB) was recently identified and characterized [59]. EgPHB showed an N-terminal hydrophobic transmembrane domain that could be involved in membrane anchorage. This EgPHB was detected in the parasite larva, germinal layer, and adult worm with overexpression in the germinal layer.

Tumor suppressor P53 has been widely studied in mammalian cells and its role in DNA repair and apoptosis is highly documented. A P53 analog (Emp53) has been identified and characterized in *E. multilocularis* [9]. While the sequence similarity with human P53 is low, Emp53 showed a DNA-binding domain with a tertiary structure similar to human P53, and it could bind to human P53 recognition sites. Authors demonstrated that increased apoptosis was induced in *E. multilocularis* when exposed to UV-C irradiation, providing evidence that Emp53 may control parasite apoptosis. Similar data are not available for *E. granulosus*, but considering the high degree of conservation of P53, further research may be successful.

Calmodulin is a protein activator that binds to many different proteins in a calcium-dependent and reversible manner [45]. Among the calmodulin-binding proteins, calcineurin has been found to be involved in apoptosis-related pathway initiation. Calmodulin was predicted as a potential drug target in *E. multilocularis* [54], and more recently identified and characterized in *E. granulosus* (Egcam) [56]. Egcam was shown to be expressed in the larva, germinal layer, and adult worm. While calmodulin is known to be involved in RCD, this was not explored in this study. The conservation and the expression of these proteins in *Echinococcus* parasites and their link with RCD subroutine was the starting point for future evidence of the role of RCD in these helminth parasites. This molecular and protein basis should be linked with the observation of apoptosis induction in cysts using medicinal and physical methods.

Naseri et al. (2016) demonstrated the pro-apoptotic effect on protoscoleces of albendazole sulfoxide and more soluble albendazole nanopolymeric particles using DNA fragmentation assays, scanning electron microscopy, and caspase-3 mRNA-expression [27]. One of the hallmarks of apoptosis and RCD is the activation of the caspase cascade, which could be detected in supernatants of activated cells. Shahnazi et al. (2017) have shown that incubation of cyst protoscoleces with an extract of 50 and 100 mg/mL of *Myrtus communis* (Myrtaceae), which has been empirically used in traditional medicine in Iran, led to caspase activation after 4 h at 37 °C [43]. Another group of researchers tested the effect of biliary acids on protoscoleces viability *in vitro* [46]. Using chenodeoxycholic acid at a concentration of 3 mM for 6 days in culture, they showed a 100% mortality rate of protoscoleces of *E. granulosus*. This effect was linked to increased caspase-3 activity.

However, in agreement with the hypothesis developed in this review, authors have tested the effect of radiotherapy for the treatment of CE in infected sheep [25]. While the same authors have previously demonstrated that X-ray treatment had a negative impact on the outcome of infected rodents [24], they tested the effect of 30, 45 and 60 Gy, divided into three doses, for 7 days on 20 infected female sheep. No significant adverse effects were detected during the 3-month follow-up. Irradiated cysts showed decreased fluid tension and partial collapse of the cystic wall. The expression of various genes during and after the treatment provided contradictory data, and no definitive conclusion can be drawn about the molecular basis of this clinical effect.

## Regulated cell death induced by the *Echinococcus* parasite

The close relationship between *Echinococcus* metacestodes and the intermediate host has been extensively described by Brehm and Koziol [6]. This contact may lead to unexpected adverse effects, such as the pro or anti-cancer properties of some components of HCF. Based on the observation that HCF activates the MAPK signaling pathway in rat liver cells, authors tested its effect on melanoma cell line A375 [16]. Using MTT assays (3-[4,5-dimethylthiazol-2,5-diphenyl tetrazolium bromide]), propidium iodide and annexin-V staining and western blotting of various RCD proteins, it was demonstrated that HCF promotes the proliferation of melanoma A375 cells, and inhibits cell apoptosis [6].

Conversely, authors have shown that a member of the Kunitz type protease inhibitor family acts as an inhibitor of neutrophil elastase and chemotaxis. This molecule (EgKI-1) has been isolated from HCF. Recombinant EgKI-1 has been produced and its activity was tested against a range of human cancer cell lines, including breast adenocarcinoma, pharynx adenocarcinoma, cervical epithelial adenocarcinoma, and melanoma [11, 38, 40]. It was shown that EgKI-1 induced apoptosis in breast cancer cells after 24 h in a dose-dependent manner [39].

## Conclusion

Although the cell death process is an answer to the question of how to determine the fate of dead cells, most of the interactions between *Echinococcus* and host tissues are associated with immune and pathologic conditions. The lack of satisfactory treatment for echinococcosis and the emergence of resistance to existing antibiotics [4, 19, 44] may raise the following question: is RCD a gateway to this parasite? Studies of the suicide genes involved in these pathways and the effects of various factors on their activity can help to identify new pharmacologic targets and improve treatment. Understanding the importance of these activations is critical because RCD is an integral part of health and disease and is stimulated by a variety of pathologic and physiologic stimuli. Histologic studies have shown that many factors are important for stimulating RCD in parasites and infected host cells [14, 35, 36]. A large number of studies have shown that the expression level of apoptosis-inducing factors in the germinal layer of sterile cysts is higher than that of fertile cysts, and the expression rate of anti-apoptotic factors in fertile cysts is higher, which may affect cystic infertility. Recently, much effort has been made to understand the mechanism of RCD and its regulation (Table 1). The recognition of cell death in different organisms and their effects on the host could pave the way to tailored therapies [20, 55]. Studying the effects of various factors on host and parasite apoptosis and identifying biochemical pathways are important steps in identifying new drug targets. Based on the current review, radiotherapy has the greatest impact on the apoptosis of hydatid cysts. Studying the pathologic pathways of cell death is also important to better understand the relationship between the host and the parasite, and the factors that alter this relationship. Factors that affect enzyme activity in hydatid cysts can be a way of inducing cystic infertility and preventing the spread of disease, particularly in the liver and lungs. The combination of these drugs may play an important role in the progressive RCD of cystic components. At the same time, hydatid cyst inhibitors that contribute to autophagy regulation have been identified, which is important to examine how the combined inputs of these regulatory circuits affect cell decisions that favor or oppose autophagy. Selective targeting of apoptosis to the germinal layer, changes in expression of apoptotic associated genes, and structural changes in various parts of the parasite can be an effective anti-echinococcosis mechanism.

**Table 1:** Cell death and host defense in hydatid cysts.

Cell death type	Effects on host-pathogen interaction	Consequences for host defense	References
Apoptosis	X-rays, carbon-ion, gamma irradiation, enhance of hydrogen peroxide and dexamethasone induces extensive DNA damage and apoptosis.	Less immune pathology	[24, 28, 30, 33, 53]
	*E. multilocularis* live worm: Up-regulation of genes that support hepatocyte apoptosis such as Gadd45c, p21, p53, and cleaved-caspase3.	Increase of immunodeficiency factors	
Necrosis	Releases of hydatic cyst fluid to extracyst space induce tissue necrosis.	Unknown	[26, 34, 58]
	Some toxic substances have scolecidal effects.		
Inflammation	IgG and IgM increase inflammation in the germinal layer and laminated layer.	Dextran sulfated sodium significantly reduces the levels of NO, IFN-γ, TNF-α and increases the production of IL-10	[13, 32, 41]
	Dextran sulfated sodium can induce acute colitis.		
Autophagy	Albendazole and Metformin can increase metacestode tissue disruption with autophagosomes.	Penetration on the laminated layer. Metacestodes from mice treated with Albendazole and Metformin showed complete tissue disruption with autophagosomes.	[22, 23, 29]
	TOR is an effective *in vitro* anti-echinococcosis agent that induces autophagy.		
	Bortezomib in the hydatid cyst can cause ER stress and provoke autophagy in protoscoleces.		
